# Correction: Hybrid predictive coding: Inferring, fast and slow

**DOI:** 10.1371/journal.pcbi.1011601

**Published:** 2023-10-27

**Authors:** The PLOS Computational Biology Staff

The first author’s name is spelled incorrectly. The correct name is: Alexander Tschantz. The correct citation is: Tschantz A, Millidge B, Seth AK, Buckley CL (2023) Hybrid predictive coding: Inferring, fast and slow. PLoS Comput Biol 19(8): e1011280. https://doi.org/10.1371/journal.pcbi.1011280. The publishers apologize for the error.
