# Supplementary material for: Hybrid predictive coding: Inferring, fast and slow
Source: PLoS Comput Biol. 2023 Aug 2;19(8):e1011280. doi: 10.1371/journal.pcbi.1011280 (PMC10395865; doi:10.1371/journal.pcbi.1011280)
Supplement: Attachment — Submitted filename: Tschantz et al review.pdf [file pcbi.1011280.s001.pdf]

Visual perception shows hallmarks of iterative inference, in that it is a process that seems to unfold over time, and not always equally quickly. This matches with the anatomical observation that the neural pathways underlying visual perception are highly recurrent. However, some aspects of visual perception seem to be nearly instantaneous, and better explained by a fast feedforward "sweep". In this new manuscript, Tschantz and colleagues propose a neural network model that attempts to reconcile these two modes of visual inference. This model builds upon the well-known "predictive coding" (PC) framework, which is fundamentally recurrent and iterative in nature, and performs inference through an often lengthy gradient descent procedure. The proposed "Hybrid Predictive Coding" (HPC) architecture extends classic recurrent PC networks with an additional feedforward network, which is trained to predict the outcome of the recurrent subnetwork's iterative inference procedure. Thus, the feedforward network provides a "shortcut" solution that can be used to initialize the recurrent computation, which can refine upon this initial guess as necessary.

The authors show that their method allows for inference that is faster than conventional PC, but more accurate than fully amortized inference. Of special interest is the fact that HPC utilizes a biologically plausible architecture and learning rules. The authors also claim that HPC retains better performance when trained on small datasets, and that HPC can flexibly invest more inference iterations to attain a desired level of accuracy on challenging or dynamically varying stimuli.

The paper is very timely and well written, and addresses an important question of interest to both neuroscientists and machine learning researchers, with mostly convincing results. However, I do have some concerns about the rationale behind, and interpretation of certain analyses, and about the conceptual framing of certain points. I have explained these concerns in more detail below.

Comments

1. Predictive coding also requires bottom-up connections

The manuscript presents the iterative, predictive coding part of the HPC architecture as being purely top-down. However, predictive coding of course requires bottom-up connections that drive updates to layer *l*'s representation as a function of the prediction error in layer *l-1*. In my opinion, the manuscript should acknowledge and clarify this in both the text and the illustrations (e.g. fig.'s 1 and 2, where the visualization implies that information flow is purely top-down in predictive coding/iterative inference).

2. Plausibility of a separate initialization network

Several recent studies have explored ways to combine iterative and amortized inference – most notably Marino et al. (2018) which coined the Iterative Amortized Inference (IAI) framework. However, at least to my knowledge, this typically involves architectures in which a single feedforward pathway continually refines estimates of the latent variables inferred by network. The current paper is somewhat of an outlier in this regard, in that it instead employs an amortized feedforward sweep through a separate network, solely for the purpose of initializing the iterative inference in a another, recurrent network (where the latter incorporates another set of bottom-up connections).

While this is a refreshing and interesting idea, it also raises some conceptual questions, especially from the point of view of emulating biology, which is one of the express purposes of this model and paper. Biological vision, after all, processes a continuous stream of input, so it is not obvious at what point it would be "initialized". How do the authors reconcile this apparent discrepancy? Do they imagine, for instance, that there is a separate computational module that decides when perception ought to be "reinitialized"? And how would such reinitializations interplay with the influence of predictions of the current state of the world derived from the immediate sensory past (which are key element of

predictive coding of time-varying input, as well as models that liken dynamic perception to a Kalman or Bayes filter)?

In any case, the initialization network would presumably play a rather minor role, being active for only brief moments, after which the bulk of neural activity plays out in the recurrent iterative inference network. How plausible is it that the brain would devote such extensive resources to a rather large subnetwork that is only used sporadically?

Continuing this line of reasoning: does it not seem more likely that the brain implements something closer to the IAI framework of Marino et al., in which a single bottom-up pathway plays the same inference-refining role at every time step? Do the authors see their present work as an intermediate step towards such a more integrated solution, or do they prefer their version with amortization "relegated" to the role of initialization? If the latter, I think the paper could be strengthened if it included some arguments for this point of view.

The above considerations do not undermine the insights gained from the paper, in my estimation, but I do feel that some discussion of these points is warranted.

3. Comparisons to amortized inference

The results presented in figures 3 and 6 draw a comparison between (hybrid) PC and amortized inference. It makes sense to include in this comparison the amortized component of the HPC network. However, to my mind there is another comparison to be drawn that is equally important – especially in the case of figure 6 – namely, to an equivalent (standalone) feedforward network trained purely discriminatively, i.e. by backpropagating a classification loss. Such a discriminatively trained network would be more representative of the most popular use of bottom-up inference, as opposed to the inversion of a generative model, such as performed by the amortized inference subnetwork of the HPC architecture.

In other words, the current results only reflect the performance of "amortized generative inference", while the analysis suggested above would reflect "amortized discriminative inference". In my opinion, the combination of both analyses would provide a fuller characterization of amortized inference and its comparison to HPC.

4. Training until convergence

The authors interpret the results presented in figure 6 as showing that HPC achieves better performance on small datasets, as compared to the amortized inference subnetwork. However, the accuracy of the amortized predictions is still apparently increasing at the point where training is terminated (after about 3000 batches). With more training, it seems possible that the amortized predictions might reach a comparable accuracy to that of the full HPC architecture. If so, then this would paint a more nuanced picture of the benefits of the HPC model. Therefore, I would like to see this analysis repeated, with the model trained until the accuracy of the amortized predictions has converged.

5. Links to variational inference

There is a well known link between predictive coding and variational inference. However, when latent variables are represented by point estimates, this link is rather trivial, as the equivalence only holds if one imagines that the variational beliefs are degenerate distributions (i.e. point masses with 0 variance). The HPC network, as implemented here, appears to fall into this latter category. As such, I disagree with the authors' characterization of their model as performing variational inference, and being "probabilistic" or employing "probabilistic representations". For instance, around l. 360, the

paper describes the iterative inference iterations as "variational iterations", around l. 537 the HPC model is described as a "probabilistic predictive coding architecture", and around l. 543 the model is said to employ "probabilistic representations". I would recommend that the paper be stripped of these characterizations, unless the authors can make a better case for why their method should be considered to be variational or probabilistic.

6. Claims about flexible inference go beyond the data

In their conclusion section, the authors write that they "demonstrate that this architecture enables both rapid and computationally cheap inference when in stable environments where a good model can be learnt, while also providing flexible, context sensitive, and more accurate inference on challenging, ambiguous, or dynamically varying stimuli" (l. 609-611 – similar claims are made at other points in the paper). This claim seems to go beyond what the results of the paper have shown. The only findings in this direction, as far as I can see, are those of fig. 7C. These results demonstrate the performance of the HPC model when a change in the data distribution occurs during training. However, I do not see how this fits the labels of inference on "challenging, ambiguous or dynamically varying stimuli". If the authors want to be able to make claims such as this, I believe this would require additional analyses in which (for instance) stimuli are systematically varied in their ambiguity or level of difficulty (however this is defined), or else a post-hoc analysis where stimuli are sorted by the number of iterations they required, and then examined for systematic patterns that predict this ranking.

7. Rationale behind fig. 7C

The rationale behind the results presented in figure 7C is not entirely clear to me. I would expect that, immediately after shifting the data distribution, the network should fail altogether to classify the stimuli, since it has never seen these new digits before (unless I misunderstand the experimental procedure). Does this failure not occur, or is it perhaps so brief as to be obscured in this plot? Or, was the predictive coding subnetwork in fact trained on a dataset with all digits, and was only the amortized subnetwork subject to a distribution shift? The latter would better explain the observed results, but not the current description in the text.

In any case, I would like to see a bit more evidence that this is more than a normal retraining effect. That is, it is to be expected that most any network, after a distribution shift, will first fail terribly and then recover its performance. But what the authors want to show here is something different, namely that the network can adaptively employ more lengthy iterative inference in order to compensate for the amortization gap that was opened by the distribution shift. Given this purpose, the most interesting moment, I would think, is immediately after the change in distribution, and before training is resumed: to what extent, exactly, can the network compensate for the novelty of the data by means of more lengthy iterative inference? The fact that, with further training, the amortization gap is gradually closed, seems less interesting to me.

8. (Apparent) typo's:

- Possible typo in equation (9): $\kappa \rightarrow \alpha$
- Typo on line 305: "Specifically, we [compute?] train and test accuracy"
