# Supplementary material for: Hybrid predictive coding: Inferring, fast and slow
Source: PLoS Comput Biol. 2023 Aug 2;19(8):e1011280. doi: 10.1371/journal.pcbi.1011280 (PMC10395865; doi:10.1371/journal.pcbi.1011280)
Supplement: Attachment — Submitted filename: review_response.pdf [file pcbi.1011280.s002.pdf]

<u>Response to Reviewers</u>

We thank the reviewers for their insightful and helpful comments and critiques, which have helped us improve the paper. Below we go through each comment individually. The original reviewer's comment is in *italics*. Our response is in **red.** The text we have added to the manuscript in response is in ***green***.

Reviewer 1

Comment 2

My other main issue concerns the description of their model. A number of important design aspects were unclear, or not easy to decipher. For example, on line 252, the authors state that they "introduce an additional set of error units". However, figure 2, which illustrates their network architecture, does not show these error nodes (although it DOES show the error nodes of the original PC network).

The amortized functions are also associated with error units used to calculate the gradients but were not visualised because figure one describes the inference process rather than learning (and these error units do not contribute to inference). We have updated the figure caption to clarify this:

"These prediction units are additionally associated with error units (not visualised) - which are not utilised for inference but are used to update the amortised parameters ϕ during learning (weight updates)."

More generally we have revisited the methodology section and added clarifications and details to make it more comprehensive. For instance, we updated our explanation of processes such as weight normalization, the use of ADAM optimizer, how entropy is calculated, and how error thresholding is performed.

Line 296:

"...a regularisation method that ensures the mean of the weights remains constant,..."

line 402:

This is achieved by normalizing the values for the final layer to obtain an output probability distribution and calculating the Shannon entropy of this distribution.

line 409:

Here, iterations are terminated when an absolute error threshold β is reached

Comment 3

Moreover, line 471 says that "the amortised and iterative inference schemes use identical connectivity and weight updates". I don't understand how the amortised and iterative inference schemes use the same weight updates. The iterative inference process uses f_theta, while the amortised inference uses f_phi. Algorithm 1 shows different weight updates for those two functions. Am I missing something? The abstract claims that "the resulting scheme can be implemented in a biologically plausible neural architecture". Without clarification of the issues above, it is difficult for me to assess that claim.

We thank the reviewer for highlighting this confusing wording. The feedforward and feedback connections are identical in their update rules. In other words, there is one learning rule, but there is functional segregation between the feedforward and feedback streams. We have updated the text to reflect this:

"Crucially, both the amortised and iterative inference schemes use the same learning rule, allowing us to posit a unified learning algorithm that underwrites both forms of inference."

Comment 4

On line 247, it says "Inference proceeds in two stages" (the amortised phase, followed by the iterative inference phase). The authors should address whether or not this two-phase design falls under the umbrella of "biologically plausible". If it does, they should elaborate on how.

We thank the reviewer for raising this point. We have added the following text to the discussion (line 560), which helps clarify the biological paradigm we are exploring, and what would need to be done in order to explore the results in other paradigms:

Empirical studies that explore the interaction between feedforward and recurrent activity typically utilize a stimulus-response paradigm, in which a single, static stimulus is introduced, and brain responses are recorded. In such paradigms, distinct feedforward and recurrent activity signatures can be observed \citep{bastos2015visual}. The present study follows this approach by conducting inference on a single, static image and simulating a feedforward pass, followed by recurrent iterations. However, the brain is exposed to continuous dynamic stimuli in natural environments, leading to a less distinct separation between feedforward and recurrent activity. In future research, we aim to expand this model to dynamic stimuli and investigate whether the hybrid predictive coding scheme can be replicated without artificially separating the two stages, such as through interleaved recurrent updates at varying temporal resolutions.

Comment 1

One of my main concerns is about its novelty. A similar model, that also included prediction connections going in both directions, was proposed inD. Xu, A. Clappison, C. Seth and J. Orchard, "Symmetric Predictive Estimator for Biologically Plausible Neural Learning," in IEEE Transactions on Neural Networks and Learning Systems, vol. 29, no. 9, pp. 4140-4151, Sept. 2018. The authors should disambiguate how their model is similar and/or different.

We thank the reviewer for bringing this work to our attention. We have added the following text to line 156:

Our work is closely related to symmetric predictive estimators (SPE) \citep{xu2017symmetric}, a model that learns separate feedforward and feedback weights using local learning rules. Similar to our work, these feedforward and feedback weights learn bottom-up and top-down projections of activity and are learned in a biologically plausible manner using local error units. Our work differs in several ways, both in technical content and theoretical motivation. In terms of technical content, the model's bidirectional connectivity is between individual neurons, rather than between layers, and the update equations have the neuron's states continually updated based on bottom-up and top-down information, rather than the temporal asymmetry found in HPC. These differences mean that the SPE does not enable the interplay of bottom-up and top-down signals of HPC. Theoretically, the SPE is not directly motivated by the Bayesian brain hypothesis and does not look to provide a common framework for feedforward and recurrent activity in the brain.

Comment 5

I don't see any reference to figure 1 in the body text.

We thank the reviewer for pointing out this omission. We have added a reference to line 109

Comment 6

line 174 says "Equation 3 introduces an additional joint distribution over hidden causes and sensory data p(z,x)". However, it is not clear to me how Eq. (3) introduces p(z,x). I would suggest the authors give a little more guidance.

We agree with the reviewer that this could be further clarified. We have added a line to equation 3 that makes the joint distribution explicit.

$$\begin{aligned}\mathcal{F}(\mathbf{z},\mathbf{x}) &= \mathbb{E}_{q_\lambda(\mathbf{z})}\big[\ln q_\lambda(\mathbf{z}) - \ln p(\mathbf{z},\mathbf{x})\big] \\ &= \mathbb{E}_{q_\lambda(\mathbf{z})}\big[\ln q_\lambda(\mathbf{z}) - \ln p(\mathbf{z}|\mathbf{x})\big] - \ln p(\mathbf{x}) \\ &\geq D_{\mathrm{KL}}\big[q_\lambda(\mathbf{z})\|p(\mathbf{z}|\mathbf{x})\big]\end{aligned} \tag{3}$$

Comment 7

line 179 says "minimising free energy with respect to theta will maximise p_theta (x) (see the second line of Equation 3)". But p(x) is not in the second line. Do the authors mean the first line?

This follows from the inequality introduced on the second (now third) line - we have removed the reference to a specific line to remove the confusion.

Comment 8

line 280: If I recall correctly, MINST has a total of 60,000 samples, which is usually split into 50,000 training samples, and 10,000 test samples.

The reviewer is correct, and we thank them for noting this error - it has been amended.

Comment 9

line 296 refers to "weight normalisation". What, precisely, is meant by that? And is it biologcially plausible?

Weight normalization is a simple form of regularisation that ensures that the mean of the weights remains constant. It may or may not be biologically plausible, but we found it necessary to maintain the accuracy of the predictive coding network (see https://arxiv.org/abs/2208.07114). It did not affect the accuracy of the hybrid predictive coding network - so we chose not to make it a focus of the current work.

We added the following clarification to the main text:

"...a regularisation method that ensures the mean of the weights remains constant,..."

Comment 10

line 299 says that the ADAM optimiser was used. That seems to contradict Algorithm 1. And how does this fit with the biological aspects of the model? A sentence or two on that would be helpful.

We thank the reviewer for this suggestion and we have added the following text to line 299:

The ADAM optimiser is treated as a generic optimiser for updating parameters via gradient descent. While the ADAM optimiser is not biologically plausible, we found that the relative results between different models identified in the paper could be recreated with alternative optimisers, such that the specific optimiser used could be treated as a black box for our key results.

Comment 11

I really like the observation that the entropy of the classification nodes decreases during iterative inference. Question: Line 298 states that the top layer does not use an activation function. So, its values need not conform to a distribution. In fact, there could be values that fall outside the [0,1] range. How do they calculate the entropy of something which likely is not a valid distribution? This should be clarified.

We have clarified our method by adding the following text to line 402:

This is achieved by normalizing the values for the final layer to obtain an output probability distribution and calculating the Shannon entropy of this distribution.

Comment 12

In addition, I am fascinated by their experiments on the number of iterations it takes to arrive at a confident inference, and what that says about the difference between a task you're learning, versus a task that you've practised and are expert on. Their allusion to the "Thinking Fast and Slow" book (by Daniel Kahneman) is apt. Given that their title is a play on Kahneman's title, perhaps it's appropriate to cite the book.

In fact, this was an inspiration for us and its omission was an oversight, we have added a reference to line 122.

Reviewer 2

Comment 1

While I agree that the model might capture some findings regarding visual processing, there are other aspects that I think are more difficult to map to biological vision. Specifically, if we take the results at face value and use HPC as a model of biological vision, we would have to conclude that we exclusively rely on feedforward processing with familiar inputs. While there is some evidence that feedback processing is less pronounced for familiar inputs, the current evidence suggests that exclusive reliance on feedforward processing is extremely unlikely to ever happen in human vision. I think this aspect needs to be addressed and discussed in more detail.

Thank you for highlighting this point. We have added the following text to 561, which we hope addresses the reviewer's concerns:

One potential criticism of the hybrid predictive coding model is that it predicts recurrent feedback activity going to zero in static environments. However, the human brain never encounters fully static data - thanks to environmental fluctuations, internal movements such as saccades - or even just ongoing internal neural fluctuations (and there is some circumstantial evidence from phenomena like retinal stabilization \citep{riggs1953disappearance,millidge2019fixational} that brain activity and perception \emph{do} go to zero for fixed stimuli). This means that amortised, feedforward predictions should never fully explain the data, so there is always a continual flow of prediction error signals that recurrent activity must explain away.

Comment 2

I don't understand how the model helps to explain visual phenomenology or how it can speak to that topic. That gist perception is based on different processes than later focussed perception is well established. One might want to claim that these different processes 'explain' different visual phenomenology. The amount of explaining that's being done is limited in my view: all that we can say is that there are different processes and that there might be different phenomenologies. Exactly how the processes lead to different phenomenologies – i.e., what the mechanisms are – remains totally unclear. But even if one accepts that the different processes of gist perception and focussed perception 'explain' phenomenology, I don't understand what the model adds.

We appreciate the reviewer bringing up this nuanced point. The claim to 'explain' phenomenology derives from the possibility that distinct modes of inference in HPC may relate to distinct of phenomenological experience, occurring under different conditions. When recapitulating the conditions in our model (i.e., different viewing times, different viewing conditions, different stimulus exposure times), we retrieve correlates within our model (i.e., the measures of perceptual certainty, relative feedforward vs. feedback activity). We claim these different correlates provide plausible accounts for their corresponding phenomenological properties. However, we recognise that these ideas remain to be experimentally tested.

We have toned down the language to reflect this position, e.g., line 29

…and may also help shed light on the neurocomputational basis of many otherwise difficult-to-understand aspects of visual phenomenology

Comment 3
Related to my two previous points: prominent theories link phenomenology to recurrent processing (as far as I understand – I'm not an expert in consciousness research). Yet, the HPC model suggests that recurrent/feedback processing diminishes as we become more familiar with a stimulus set up to the point where vision is entirely based on feedforward processing. Thus, there seems to be a discrepancy between claims in the literature about feedback processing and phenomenology, and the claims made in this paper. According to HPC, if feedback processing determines phenomenology, then we should see a change in phenomenology as we become more familiar with a stimulus.

We have added the following text to 561, which highlights the idea that full familiarity will never occur due to the dynamic nature of stimuli (and sensors) in natural environments. However, it is our claim that phenomenology changes as a function of familiarity with a stimulus - e.g., in gist perception (a similar argument may be made for early development during infancy, although we do not make this argument in the paper).

One potential criticism of the hybrid predictive coding model is that it predicts recurrent feedback activity going to zero in static environments. However, the human brain never encounters fully static data - thanks to environmental fluctuations, internal movements such as saccades - or even just ongoing internal neural fluctuations (and there is some

circumstantial evidence from phenomena like retinal stabilization \citep{riggs1953disappearance,millidge2019fixational} that brain activity and perception \emph{do} go to zero for fixed stimuli). This means that amortised, feedforward predictions should never fully explain the data, so there is always a continual flow of prediction error signals that recurrent activity must explain away.

Comment 4

I would tone down the claims regarding the neurophysiological plausibility of the model (throughout the whole paper). I'm not convinced that the model is more (or less) neurophysiologically plausible than many other models that have tried to address a similar issue.

We have clarified the view by adding the following text to line 244. All other claims of biological plausibility reference predictive coding directly, rather than hybrid predictive coding, to ensure that there is no confusion (i.e. that we are claiming our model is more plausible than PC):

Claims of biological plausibility inherit from the predictive coding framework, which adheres to the principles of local computation and local plasticity \citep{rao_predictive_1999,buckley2017free,bogacz2017tutorial,millidge2021predictive}. We do not claim this model to be any more (or less) biologically plausible than networks that meet these criteria. However, we do claim that our models have an increased degree of biological relevance, due to the fact that they can account for empirical observations that evade explanation in a traditional predictive coding framework.

Comment 5

I would tone down some of the claims in the discussion/conclusion section. For instance, you say: 'Hybrid predictive coding offers a new perspective on the biological relevance of the feedforward sweeps and iterative recurrent activity…' I don't think that's true. There are many proposals – both conceptual and empirical – that have made similar suggestions before. In fact, in other parts of the paper, you use these other proposals to claim that 'This account of perception is remarkably consistent with our proposed model.' I think it would be more accurate to say that you provide a new model that captures old perspectives.

We agree with the reviewer that the contribution of the work is a synthesis of existing ideas rather than proposing novel perspectives. We thank them for their input and have updated that particular text to read (line 619):

Hybrid predictive coding thus provides a novel model that unifies disparate perspectives on the biological role of feedforward sweeps and recurrent activity in the visual cortex,

offering a parsimonious explanation for several different and often opposing perspectives

Comment 6

Page 8: You say that 'Equation 3 introduces an additional joint distribution over hidden causes and sensory data p(z, x)…'. However, Equation 3 doesn't introduce that joint distribution. It's therefore not clear what you are referring to, which is confusing for the reader (or at least it was for me).

We thank the reviewer for noting this point of confusion. We have added a line to equation 3 that makes the joint distribution explicit.

$$\begin{aligned} \mathcal{F}(\mathbf{z},\mathbf{x}) &= \mathbb{E}_{q_\lambda(\mathbf{z})}\big[\ln q_\lambda(\mathbf{z}) - \ln p(\mathbf{z},\mathbf{x})\big] \\ &= \mathbb{E}_{q_\lambda(\mathbf{z})}\big[\ln q_\lambda(\mathbf{z}) - \ln p(\mathbf{z}|\mathbf{x})\big] - \ln p(\mathbf{x}) \\ &\geq D_{\mathrm{KL}}\big[q_\lambda(\mathbf{z})\|p(\mathbf{z}|\mathbf{x})\big] \end{aligned} \tag{3}$$

Comment 7

Page 14: More detail about the simulations are needed. Specifically, how do you assess accuracy of the amortized component over batches? As far as I understand what you do is this: 1. perform the amortized initial best guess and log that best guess as this component's accuracy for the current input; 2. Refine the guess via the top-down component of the model and use the refined inference to update the amortized parameters. 3. Use the updated amortized parameters for the next input, and start with 1. Is that correct? If so, I think it would be worth spelling this out explicitly. In any case, in my view, it would be beneficial to be more explicit about exactly what you have done here.

This is correct; we thank the reviewer for highlighting the ambiguity and we have clarified the procedure on line 319:

We calculate amortised accuracy by an amortised feedforward sweep and using the resulting activations of the final layer to calculate the loss, and the amortised model is then updated based on the bottom-up errors between results from this initial feedforward sweep and the final activation derived from iterative processing.

Comment 8

Fig. 3 A: It's worth highlighting and explaining why the standard PC model initially performs stronger than the HPC model but seems to show reduction in performance with increasing batch numbers.

We thank the reviewer for pointing out this unintuitive result and have added the following text to 352:

As illustrated in Figure \ref{fig:6}, the predictive coding and hybrid predictive coding architectures exhibit the capacity to learn from limited data samples, which may account for the superior performance of the predictive coding network during initial training stages. Nevertheless, the predictive coding network's accuracy shows a marginal decline due to training procedure instabilities absent in hybrid predictive coding.

We have further explained this result on line 374:

We identify several potentially counterintuitive results that require further explanation, and which may motivate future empirical explorations. First, the accuracy of the amortised network improves slower as the number of iterative iterations increases. We hypothesise that this is due to the targets for the amortised network being more difficult to predict (due to an increased number of iterations, leading to increased accuracy). Second, the amortised component can achieve high accuracy while the iterative component achieves low accuracy. This is due to the final layer loss - which is a function of the ground truth labels and which provides a learning signal in lieu of accurate iterative inference. Finally, the instabilities in the predictive coding network occur at a low number of iterations, leading to a decline in accuracy. We hypothesize that these result from instabilities in the training procedure, which crucially are not found in the fully hybrid network \citep{kinghorn2022preventing}.

Comment 9

9. Fig. 5 features several aspects that are a bit confusing (for my brain at least) but that are not highlighted or discussed: 1. The amortized component of the HPC model seems to perform better with fewer iterations. For instance, after 600 batches, it's accuracy is at around 0.6 with 10 iterations but at around 0.5 with 100 iterations. Why does this happen? I find it difficult to get my head around it.
2. The HPC model seems to perform equally well independently of the number of iterations. How is that possible given that the generative component on its own struggles with the lower number of iterations? I assume that the amortized component compensates the difficulties of the generative component (which you kind of allude to). However, relating to my previous point, I find it difficult to see how the amortized component is able to perform better on low iterations (in particular, given that it's updating is dependent on the accuracy of the generative component). 3. The standard PC shows interesting behavior with 25 and 50 iterations. It might be useful to highlight and explain both the better performance compared to HPC early on and the breakdown after more batches (also see my previous point).

We hypothesize that (1) is due to the target for the amortised network being harder to predict, due to an increased number of iterations and increased accuracy of the iterative network). In other words, the function that the amortised model needs to learn is more

complex in the case where iterative inference is applied. For (2), this is correct, the amortized component compensates for the lack of iterations by providing a feedforward estimate of parameters. The discrepancy noted is in part due to the final layer loss, which is between the true labels and the activations - and thus not a direct function of the iterative procedure. We hypothesize that (3) is related to training instabilities in predictive coding networks, see https://arxiv.org/abs/2208.07114. To better explain the above points ,we have added the following text to line 374:

We identify several potentially counterintuitive results that require further explanation, and which may motivate future empirical explorations. First, the accuracy of the amortised network improves slower as the number of iterative iterations increases. We hypothesise that this is due to the targets for the amortised network being more difficult to predict (due to an increased number of iterations, leading to increased accuracy). Second, the amortised component can achieve high accuracy while the iterative component achieves low accuracy. This is due to the final layer loss - which is a function of the ground truth labels and which provides a learning signal in lieu of accurate iterative inference. Finally, the instabilities in the predictive coding network occur at a low number of iterations, leading to a decline in accuracy. We hypothesize that these result from instabilities in the training procedure, which crucially are not found in the fully hybrid network \citep{kinghorn2022preventing}.

Comment 10

Page 19: I'm not quite sure I understand why the learning with limited data isn't shown for the classic PC model.

We thank the reviewer for their valuable and valid comment. This figure was designed to demonstrate the relative difference between iterative inference and amortized inference concerning data efficiency - and as such we have selected two models to capture these traits. However, demonstrating that predictive coding is data-efficient would help highlight that it is this component of the model that lends hybrid predictive coding to these traits.

Comment 11

1. I think you might want to reference Moshe Bar's work more explicitly in the introduction. His proposal is one of the first that is very similar to what you suggest here in terms of the role of feedforward and feedback processing.

Thank you for highlighting this work, it was indeed an omission it was not referenced. We have included reference to their work in the introduction when introducing the relevant concepts in the introduction [1].

[1] Bar, M. (2003). A cortical mechanism for triggering top-down facilitation in visual object recognition. Journal of cognitive neuroscience, 15(4), 600-609.

Comment 12

2. Page 4: I don't quite understand this sentence: 'Although classical bottom-up perspectives are often contrasted with Bayesian top-down theories in this debate, more nuanced pictures have also been proposed in which bottom-up and top-down signals both contribute to perceptual content, but in distinct ways.' The original predictive coding (and similar) accounts also suggest that both bottom-up and top-down signals contribute to perceptual content. I think it would be useful to be more specific about what the cited proposals add.

While the original PC objective did utilize both bottom-up and top-down information flows, this is in the service of performing a single round of iterative inference on the stimulus where the bottom-up flows are necessary to convey information up the hierarchy. Our model, in contrast, adds an entirely new type of amortized inference into the model in addition to the bottom-up flows already inherent within the predictive coding framework. Crucially, the bottom-up amortized sweep in our model enables an immediate initialization of variables throughout the hierarchy to their amortized predictions – enabling an extremely rapid transfer of information – while in standard PC the bottom-up information slowly percolates upwards through gradient dynamics.

We have noted relevant manuscript changes to the related comment below.

3. Page 4: Related to the previous point, it might be useful to be more specific here: 'Here, we develop, and illustrate with simulations, a novel computational architecture in which top-down and bottom-up signalling is adaptively combined to bring about perceptual representations within an extended predictive coding paradigm.' As I said, bottom-up and top-down signals are combined in most accounts of perceptual processing. What's special about your account? I think the key novelty of your proposal is the idea that predictions are not only implemented in top-down processing but integrated into bottom-up processing (as conceptually suggested by Teufel & Fletcher 2020).

We thank the reviewer for highlighting this point and agree with their assessment. We have clarified our claims with the following text:

In the caption of Figure 1:

Although there is bottom-up information flow [in PC] in terms of errors, this bottom-up information only conveys errors. However, in our hybrid predictive coding models, bottom-up information conveys both predictions and errors (along with top-down information).

We have added the following text to line 117:
This contrasts with traditional predictive coding, where bottom-up signals only convey errors, and bottom-up connectivity does not implement transformations between layers (i.e. does not implement predictions).

Comment 14
4. Page 4: 'In this setting, bottom-up signals convey prediction errors - the difference between predictions and data - whereas top-down signals convey the predictions.' It might be worth highlighting that not all PC schemes work this way (e.g., Michael Spratling's work).

Thank you for raising this extremely relevant point, we have included a reference on line 81:
"although see alternative predictive coding schemes which depart from this template \citep{spratling2017review}, although in different ways to our proposed hybrid predictive coding model."

Comment 15
5. Page 7: '…first to combine of iterative and amortized inference…' Get rid of the 'of'
We thank the reviewer for finding this typo and have updated the main text.

Comment 16
6. Page 8: '…bound on Eq. 2, i.e. a quantity which…' a comma is missing after 'i.e.'
This error has been updated in the main text.

Comment 17
7. Page 8: '…parameters θ, e.g pθ(z, x).' comma and full stop are missing after 'e.g'. In fact, the missing comma after 'i.e.' and e.g.' is a more general mistake throughout the paper.
We have been through and updated all instances of i.e., and e.g., - we thank the reviewer for highlighting these errors.

Comment 18
8. Page 10: '…here denoted μ∗…' I would say: '… denoted μ∗ in considerations of HPC below…'.

We have updated the main text with this suggestion.

Comment 19

9. Page 10: This is a bit confusing '…going from data (stimuli) to hidden states (features)…' as you previously used the term 'features' akin to 'data' when you say that predictive coding predicts features from objects.

We agree that this is confusing terminology and have updated the phrase to "latent" features in the main text.

Comment 20

10. Page 13: I would use 'Learning of Amortized Parameters' rather than just 'Learning' to avoid ambiguity in the illustration.

We thank the reviewer for highlighting this clarification and have updated the main text.

Comment 21

11. Page 13: I would suggest you start line 252 with something like: 'Once inference is completed for the current input, generative and amortized parameters are updated via learning.'

We agree that this makes the narrative easier to understand. We have included this suggestion and are grateful to the reviewer for pointing this out.

Comment 22

12. Page 19: '…incorporating a bottom, amortised component…' It should be 'bottom-up' rather than 'bottom'

We have updated this typo and appreciate the reviewer raising this.

Comment 23

13. Page 19: 'Note THAT we still use the complete test set of 10,000 images for testing.'

This typo has been corrected.

Comment 24

14. Page 19: 'To gain intuition further intuition…' get rid of the first 'intuition'

We apologize for this typo and have corrected the main text.

Comment 25*15. Page 21: Fig. 7B: I assume you used a stopping criterion here (i.e., the number of iterations required to reach a specific criterion)? I might have missed it but I think that might not be specified anywhere.*

We thank you the reviewer for pointing out this omission and have added to line 409:
Here, iterations are terminated when an absolute error threshold β is reached

Reviewer 3

Comment 1
The manuscript presents the iterative, predictive coding part of the HPC architecture as being purely top-down. However, predictive coding of course requires bottom-up connections that drive updates to layer l's representation as a function of the prediction error in layer l-1. In my opinion, the manuscript should acknowledge and clarify this in both the text and the illustrations (e.g. fig.'s 1 and 2, where the visualization implies that information flow is purely top-down in predictive coding/iterative inference).

We thank the reviewer for pointing out this clarification - we have clarified that the role of bottom-up (and top-down) connectivity and activity distinguishes the two frameworks.

We have added the following text to the caption of Figure 1:
Although there is bottom-up information flow [in PC] in terms of errors, this bottom-up information only conveys errors. However, in our hybrid predictive coding models, bottom-up information conveys both predictions and errors (along with top-down information).

We have added the following text to line 117:
This contrasts with traditional predictive coding, where bottom-up signals only convey errors, and bottom-up connectivity does not implement transformations between layers (i.e. does not implement predictions).

Comment 2
Several recent studies have explored ways to combine iterative and amortized inference – most notably Marino et al. (2018) which coined the Iterative Amortized Inference (IAI) framework. However, at least to my knowledge, this typically involves architectures in which a single feedforward pathway continually refines estimates of the latent variables inferred by network.

The current paper is somewhat of an outlier in this regard, in that it instead employs an amortized feedforward sweep through a separate network, solely for the purpose of initializing the iterative inference in a another, recurrent network (where the latter incorporates another set of bottom-up connections). While this is a refreshing and interesting idea, it also raises some conceptual questions, especially from the point of view of emulating biology, which is one of the express purposes of this model and paper. Biological vision, after all, processes a continuous stream of input, so it is not obvious at what point it would be "initialized". How do the authors reconcile this apparent discrepancy? Do they imagine, for instance, that there is a separate computational module that decides when perception ought to be "reinitialized"? And how would such reinitializations interplay with the influence of predictions of the current state of the world derived from the immediate sensory past (which are key element of predictive coding of time-varying input, as well as models that liken dynamic perception to a Kalman or Bayes filter)? In any case, the initialization network would presumably play a rather minor role, being active for only brief moments, after which the bulk of neural activity plays out in the recurrent iterative inference network. How plausible is it that the brain would devote such extensive resources to a rather large subnetwork that is only used sporadically?
Continuing this line of reasoning: does it not seem more likely that the brain implements something closer to the IAI framework of Marino et al., in which a single bottom-up pathway plays the same inference-refining role at every time step? Do the authors see their present work as an intermediate step towards such a more integrated solution, or do they prefer their version with amortization "relegated" to the role of initialization? If the latter, I think the paper could be strengthened if it included some arguments for this point of view.
The above considerations do not undermine the insights gained from the paper, in my estimation, but I do feel that some discussion of these points is warranted.

Thank you for your in-depth comments, this is a crucial area that certainly warrants further exploration. The difference between our work and that of Marino (on the current topic - the type of model and precise functionals also differ) arises from the number of iterative steps that occur after a stimulus is presented (i.e., after an amortised forward sweep). If we were to set the number of iterations to one, the models would be equivalent in this regard. We believe this collapses the question about the interplays between feedforward and feedback connections. We have made the first steps in exploring how these can be automatically modulated, e.g., with the iterative inference threshold experiments. In future work, we hope to explore how parameters of the generative model (such as precisions) can provide further dynamic modulation between these two streams of information and explore how this might relate to cognitive attention. To address the biological/computational questions regarding separate feedforward and recurrent stages, have added the following text to the discussion (line 560).

Empirical studies that explore the interaction between feedforward and recurrent activity typically utilize a stimulus-response paradigm, in which a single, static stimulus is introduced, and brain responses are recorded. In such paradigms, distinct feedforward and recurrent activity signatures can be observed \citep{bastos2015visual}. The present

study follows this approach by conducting inference on a single, static image and simulating a feedforward pass, followed by recurrent iterations. However, the brain is exposed to continuous dynamic stimuli in natural environments, leading to a less distinct separation between feedforward and recurrent activity. In future research, we aim to expand this model to dynamic stimuli and investigate whether the hybrid predictive coding scheme can be replicated without artificially separating the two stages, such as through interleaved recurrent updates at varying temporal resolutions.

Comment 3

The results presented in figures 3 and 6 draw a comparison between (hybrid) PC and amortized inference. It makes sense to include in this comparison the amortized component of the HPC network. However, to my mind there is another comparison to be drawn that is equally important – especially in the case of figure 6 – namely, to an equivalent (standalone) feedforward network trained purely discriminatively, i.e. by backpropagating a classification loss. Such a discriminatively trained network would be more representative of the most popular use of bottom-up inference, as opposed to the inversion of a generative model, such as performed by the amortized inference subnetwork of the HPC architecture.

In other words, the current results only reflect the performance of “amortized generative inference”, while the analysis suggested above would reflect “amortized discriminative inference”. In my opinion, the combination of both analyses would provide a fuller characterization of amortized inference and its comparison to HPC.

This is would indeed be an interesting comparison. We did not consider backpropagation due to its violation of the biologically plausible constraints set out (local computation + local plasticity). We believe that such models fall outside the scope of the paper's wish to explore, which is the design space of feedforward/feedback networks prefaced on predictive coding. However, we expect backpropagation algorithms to perform similarly to the amortised model, in terms of data efficiency, albeit with higher asymptotic performance due to how errors are propagated.

Comment 4

The authors interpret the results presented in figure 6 as showing that HPC achieves better performance on small datasets, as compared to the amortized inference subnetwork. However, the accuracy of the amortized predictions is still apparently increasing at the point where training is terminated (after about 3000 batches). With more training, it seems possible that the amortized predictions might reach a comparable accuracy to that of the full HPC architecture. If so, then this would paint a more nuanced picture of the benefits of the HPC model. Therefore, I would like to see this analysis repeated, with the model trained until the accuracy of the amortized predictions has converged.

We thank the reviewer for their insightful comment. The purpose of this experiment is to compare the relative times to asymptote, rather than absolute performance. Thus a stopping criterion is used for when one of the two networks stops improving. We explore asymptotic performance in Figure 3a, where we explore the asymptotic performance of the networks (i.e., run them all to convergence). Indeed, as predicted, the amortised predictions do reach the full accuracy of the predictive coding and hybrid predictive coding networks.

Comment 5

There is a well known link between predictive coding and variational inference. However, when latent variables are represented by point estimates, this link is rather trivial, as the equivalence only holds if one imagines that the variational beliefs are degenerate distributions (i.e. point masses with 0 variance). The HPC network, as implemented here, appears to fall into this latter category. As such, I disagree with the authors' characterization of their model as performing variational inference, and being "probabilistic" or employing "probabilistic representations". For instance, around l. 360, the paper describes the iterative inference iterations as "variational iterations", around l. 537 the HPC model is described as a "probabilistic predictive coding architecture", and around l. 543 the model is said to employ "probabilistic representations". I would recommend that the paper be stripped of these characterizations, unless the authors can make a better case for why their method should be considered to be variational or probabilistic.

We take the point that predictive coding, in the current setting, shares many features with a deterministic system. However, we prefer to keep the current terminology as it is ubiquitous in the predictive coding literature to describe it as probabilistic due to the fact it is motivated by and derived from variational inference. The point mass distributions on the approximate posterior can be motivated by the Laplace approximation [1]. Moreover, the likelihoods are still treated as Multivariate Gaussian distributions, albeit with fixed second-order statistics. We have added the following text to the manuscript to clarify (line 212)

It is for this reason that predictive coding is often considered a `probabilistic` theory of learning (as the model variables can be read as sufficient statistics of posterior beliefs), even when all of the distributions are point mass or with fixed second-order statistics.

[1] Buckley, C. L., Kim, C. S., McGregor, S., & Seth, A. K. (2017). The free energy principle for action and perception: A mathematical review. *Journal of Mathematical Psychology*, *81*, 55-79.

Comment 6

In their conclusion section, the authors write that they "demonstrate that this architecture enables both rapid and computationally cheap inference when in stable environments where a good model can be learnt, while also providing flexible, context sensitive, and more accurate inference on challenging, ambiguous, or dynamically varying stimuli" (l. 609-611 – similar claims are made at other points in the paper). This claim seems to go beyond what the results of the paper have shown. The only findings in this direction, as far as I can see, are those of fig. 7C. These results demonstrate the performance of the HPC model when a change in the data distribution occurs during training. However, I do not see how this fits the labels of inference on "challenging, ambiguous or dynamically varying stimuli". If the authors want to be able to make claims such as this, I believe this would require additional analyses in which (for instance) stimuli are systematically varied in their ambiguity or level of difficulty (however this is defined), or else a post-hoc analysis where stimuli are sorted by the number of iterations they required, and then examined for systematic patterns that predict this ranking.

Thank you for raising this important clarification. We have updated the text to reflect the conclusions directly implied by the experiments more accurately:

We demonstrate that this architecture enables both rapid and computationally cheap inference when in stable environments where a good model can be learned, while also flexibly providing more accurate inference in sparse data or early learning regimes. The results of our model additionally suggest that hybrid predictive coding will behave favorably when faced with more difficult stimuli, due to it being able to flexibly deploy additional computation as a function of experience with a given stimulus

Comment 7

The rationale behind the results presented in figure 7C is not entirely clear to me. I would expect that, immediately after shifting the data distribution, the network should fail altogether to classify the stimuli, since it has never seen these new digits before (unless I misunderstand the experimental procedure). Does this failure not occur, or is it perhaps so brief as to be obscured in this plot? Or, was the predictive coding subnetwork in fact trained on a dataset with all digits, and was only the amortized subnetwork subject to a distribution shift? The latter would better explain the observed results, but not the current description in the text.

In any case, I would like to see a bit more evidence that this is more than a normal retraining effect. That is, it is to be expected that most any network, after a distribution shift, will first fail terribly and then recover its performance. But what the authors want to show here is something different, namely that the network can adaptively employ more lengthy iterative inference in order to compensate for the amortization gap that was opened by the distribution shift. Given this purpose, the most interesting moment, I would think, is immediately after the change in distribution, and before training is resumed: to what extent, exactly, can the network compensate for the novelty of the data by means of more lengthy iterative inference? The fact that, with further training, the amortization gap is gradually closed, seems less interesting to me.

We thank the reviewer for raising this extremely relevant point. We believe that the requested data can be determined from the existing manuscript and figures. Directly after the distribution shift, we run inference (and thus no learning has happened since the distribution shift). The point on the x-axis directly after the distribution shift, therefore, signifies the number of iterative iterations, which jump from ~1 before the shift to ~100 after the shift, with no further retraining (for that particular point). The rest of the graph shows how the number of iterations decreases as the system fits the new distribution. We believe that this is different from the amortization gap, which would simply show the final loss before and after the distribution shift, rather than the number of iterations employed. Indeed, after the distribution shifts the network does fail to classify (not shown in figures), resulting in larger prediction errors after the amortized sweep, which incurs more recurrent processing. This figure is helpful to consider in combination with 7b, which shows that vanilla predictive coding does not induce significantly reduced iterations as a function of learning (due to the network being randomly initialized for each stimulus) - so the number of iterations does not track familiarity with the data distribution.

Comment 8

Possible typo in equation (9): $\kappa \rightarrow \alpha$

We thank the reviewer for spotting this typo and have updated the text.

- *Typo on line 305: “Specifically, we [compute?] train and test accuracy”*

We apologize for missing this word and have updated the text accordingly.
