# Supplementary material for: Hybrid predictive coding: Inferring, fast and slow
Source: PLoS Comput Biol. 2023 Aug 2;19(8):e1011280. doi: 10.1371/journal.pcbi.1011280 (PMC10395865; doi:10.1371/journal.pcbi.1011280)
Supplement: Attachment — Submitted filename: Tschantz et al revision review.pdf [file pcbi.1011280.s003.pdf]

In this revised manuscript, Tschantz et al. partly address the concerns I raised in my previous review. I thank them for the revisions they have made, and I want to stress that I remain very enthusiastic about the work in general, and especially the ideas behind it. However, I also still have strong reservations about some aspects of how the work is presented. These reservations concern (1) a misrepresentation of the role of bottom-up connections in “vanilla” predictive coding models as compared to the HPC model, (2) misguided and misleading (and, indeed, unnecessary) claims about the (putative) probabilistic or variational nature of the HPC model’s computations and representations and (3) a misrepresentation of results regarding data efficiency. While these concerns can be addressed with rather minor revisions or additional analyses, I do find them to be of high importance for the paper’s scientific soundness.

For clarity, I have explained my concerns in detail below, as responses to the replies made by the authors to my earlier points.

Comment 1

We thank the reviewer for pointing out this clarification - we have clarified that the role of bottom-up (and top-down) connectivity and activity distinguishes the two frameworks.

We have added the following text to the caption of Figure 1:

Although there is bottom-up information flow [in PC] in terms of errors, this bottom-up information only conveys errors. However, in our hybrid predictive coding models, bottom-up information conveys both predictions and errors (along with top-down information).

We have added the following text to line 117:

This contrasts with traditional predictive coding, where bottom-up signals only convey errors, and bottom-up connectivity does not implement transformations between layers (i.e. does not implement predictions).

> I appreciate these revisions, and I feel that figure 1 is now sufficiently clear. However, I still have reservations about figure 2 and the description in the text.
>
> In their revised text, the authors write that, in traditional PC, “bottom-up connectivity does not implement transformations between layers” and “bottom-up signals only convey errors”. But said errors of course are multiplied by synaptic weights in order to drive updates to the representation units in the layer above. This multiplication (and subsequent summation) surely constitutes a transformation, and this is fundamentally no different from the bottom-up transformations that occur in the amortized component of the hybrid PC model. In the latter, the activities of the representation units are multiplied by synaptic weights to directly yield a prediction of the activities of the representation units above. Both constitute a linear transformation of an activity vector (combined with a pointwise non-linearity). So, the difference is not that one is a transformation while the other isn’t – the difference is in what each transformation accomplishes. One projects prediction errors onto the Jacobian of the (local) generative model, while the other projects the mu’s onto an approximate inverse of that generative model. In both cases, the bottom-up synaptic weight matrix has the role of transforming a lower-level representation into a higher, more abstract one. Thus, the current

phrasing is doing a disservice to traditional PC models, and does not accurately explain the contribution of the amortized bottom-up stream that is added in the HPC model.

As for figure 2, my issue with this is that there are no bottom-up arrows from the prediction errors to the mu's in the layer above, and I really feel there should be (especially since there *are* arrows from the errors going down to the mu's in the layer below, even though these arrows represent the same functionality and connectivity that is omitted in the bottom-up direction). In its current design, the figure incorrectly suggests that the iterative inference is purely top-down, and that errors only drive updates to the layer directly below them.

Comment 4

We thank the reviewer for their insightful comment. The purpose of this experiment is to compare the relative times to asymptote, rather than absolute performance. Thus a stopping criterion is used for when one of the two networks stops improving. We explore asymptotic performance in Figure 3a, where we explore the asymptotic performance of the networks (i.e., run them all to convergence). Indeed, as predicted, the amortised predictions do reach the full accuracy of the predictive coding and hybrid predictive coding networks.

If the purpose was to compare the speed of training, then this analysis does indeed make more sense. However, speed of training (or time to asymptote) is not a measure of data efficiency – a model can train very slowly and yet be very data-efficient. More to the point, if the amortized component of the HPC model eventually converged to a similar performance as the full HPC model, then both would be equally data-efficient. So, at present, the claims made on this point (e.g., "These results show that incorporating a top-down, generative component substantially **increases data efficiency**." (l. 426-427)) are not substantiated by the data. The simplest way to address this concern would be (as I suggested previously) to compare asymptotic performance in the low-data regime. If the authors are unable to do this comparison, then I feel they should revise the claims made in the manuscript to be about speed of training, rather than data efficiency.

In addition, if the authors prefer to stick with an analysis of training speed, then I think they need to revise some of their phrasing in the text, which implies that their present results constitute a comparison of accuracy *per se*. For instance (emphasis mine):

"Here, we demonstrate this data-efficiency by plotting accuracy as a function of dataset size. Specifically, we compare the accuracy of hybrid predictive coding compared to the amortised predictions using datasets with 100, 500, 1000 and 5000 examples" (l. 420-422)

"Figure 6: Accuracy as a function of dataset size. (A) 100 examples. The accuracy of hybrid predictive coding is lower than with the full dataset, but still high given the minimal amount of data (0.17 percent). The accuracy of the amortised predictions is significantly worse." (Fig. 6 caption)

Comment 5

We take the point that predictive coding, in the current setting, shares many features with a deterministic system. However, we prefer to keep the current terminology as it is ubiquitous in the predictive coding literature to describe it as probabilistic due to

the fact it is motivated by and derived from variational inference. The point mass distributions on the approximate posterior can be motivated by the Laplace approximation [1]. Moreover, the likelihoods are still treated as Multivariate Gaussian distributions, albeit with fixed second-order statistics. We have added the following text to the manuscript to clarify (line 212)

It is for this reason that predictive coding is often considered a `probabilistic` theory of learning (as the model variables can be read as sufficient statistics of posterior beliefs), even when all of the distributions are point mass or with fixed second-order statistics.

I do not feel that this addition addresses my concern, and I disagree with the authors' reasoning and assertions.

Predictive coding was not originally derived from variational inference, and does not uniquely derive from this framework. As the authors are no doubt aware, one can derive predictive coding simply from a mean-squared-error (MSE) loss, or from maximum-likelihood (ML) or maximum-a-posteriori estimation (MAP) on a hierarchical Gaussian generative model. Optimizing a MSE loss can be seen as a special case of ML or MAP estimation (by assuming IID Gaussian noise at all levels, combined with a flat prior), just as ML and MAP estimation can in turn be seen as a special case of variational inference (if the variational beliefs are set to be point masses). However, while these connections are insightful, it is misleading to refer to each special case by its parent category, just as it would be misleading to refer to a parabola as a 10-D polynomial (even though the former is technically a special case of the latter). Using the more general label implies the additional properties that distinguish the special from the general case (for lack of them).

PC models can, and have been, extended or modified to perform variational inference – that is, to represent variables by non-trivial probability distributions that approximate the true posterior. But HPC is not such a model, and PC models are not variational by default. (There is a reason why VAEs are called "variational" in contrast to regular autoencoders. By the logic put forth by the authors, *all* autoencoders would be VAEs, and indeed, all linear regression analyses would be variational too, and so on.)

The Laplace approximation can of course be used to estimate an approximate posterior around a MAP estimate, but the method presented here does not employ such an approximation. So, just as one should not market an automobile as an aircraft because one *could* affix wings to it, the HPC method should not be called "probabilistic" merely because one *might* combine it with a Laplace approximation.

To clarify, I have no objections to calling the generative model "probabilistic", to pointing out that PC can be thought of as performing inference on a PGM, or to explaining the relationship between (H)PC and variational inference. However, to refer to the representations computed by the HPC inference process (or that inference process itself) as "probabilistic", or to refer to any aspect of the HPC model as "variational" (e.g., "variational iterations"), is to stretch these terms beyond their breaking point, and to imply that the model has abilities that it does not possess.

(See also Ma, W. J. (2012). Organizing probabilistic models of perception. *Trends in cognitive sciences, 16*(10), 511-518.)

Comment 7

We thank the reviewer for raising this extremely relevant point. We believe that the requested data can be determined from the existing manuscript and figures. Directly after the distribution shift, we run inference (and thus no learning has happened since the distribution shift). The point on the x-axis directly after the distribution shift, therefore, signifies the number of iterative iterations, which jump from ~1 before the shift to ~100 after the shift, with no further retraining (for that particular point). The rest of the graph shows how the number of iterations decreases as the system fits the new distribution. We believe that this is different from the amortization gap, which would simply show the final loss before and after the distribution shift, rather than the number of iterations employed. Indeed, after the distribution shifts the network does fail to classify (not shown in figures), resulting in larger prediction errors after the amortized sweep, which incurs more recurrent processing. This figure is helpful to consider in combination with 7b, which shows that vanilla predictive coding does not induce significantly reduced iterations as a function of learning (due to the network being randomly initialized for each stimulus) - so the number of iterations does not track familiarity with the data distribution.

Thank you for providing this explanation. I think I am beginning to understand, although it would be helpful (also to other readers, I suspect) if you could fill in a few more gaps for me. My confusion stemmed from the assumption that the network should never reach the threshold level of perceptual certainty, when faced with a digit from an unfamiliar class (e.g. an '8' when only 0-4 were seen during training). However, I now surmise that this intuition was incorrect, and that the network nevertheless converges to some sufficiently confident (but most likely incorrect) guess about the digit class. Is this understanding correct? I guess this must depend on the choice of threshold: with a sufficiently low threshold, I would expect my initial prediction to be confirmed (at least in some instances). Perhaps it would be useful to plot the classification accuracy along the number of iterations, to show that there is nothing "magical" going on (i.e. the model cannot classify digit classes it never saw before).

I think this is worth noting because it means that, while the network is able to converge to some answer with sufficient iterations, no amount of iterative processing is sufficient to accurately classify digits from unfamiliar classes.

My confusion was also specifically fed by the following passage:

"As 7C shows, this dramatically increases the number of iterations required to reach perceptual certainty. This is because the bottom-up, amortised predictions have not learned the predict the relevant model variables, leading to a poor initialisation and an increase in prediction error."

I would have thought that the top-down predictions would be just as inaccurate (at least at higher levels of representation, nearer the digit class units) as the bottom-up, amortized predictions. After all, the network has not (if I understand correctly) had the chance to learn a generative model of the unseen digit classes (though these digits of course share lower-level features in common that will have been learned). Is this not the case, or does this not contribute to the increased number of required iterations?

(The wording of this passage initially led me to think that perhaps the generative model (i.e. the top-down connections) *had* been trained on the withheld digit classes, thus enabling the iterative inference to accurately classify the digit, only without the benefit of a good initialization.)
