# Supplementary material for: Hybrid predictive coding: Inferring, fast and slow
Source: PLoS Comput Biol. 2023 Aug 2;19(8):e1011280. doi: 10.1371/journal.pcbi.1011280 (PMC10395865; doi:10.1371/journal.pcbi.1011280)
Supplement: Attachment — Submitted filename: PLOSCB R2 RESPONSE.pdf [file pcbi.1011280.s004.pdf]

PLoS CB HPC paper
Second revisions

May 2, 2023

We thank the reviewers for their continued insightful and helpful comments and critiques on the revised version of our paper. We believe we have addressed all the comments, and we hope the new revision will be considered satisfactory. Below we go through each comment individually. The original reviewer's comment is in *italics*. Our response is in **red.** The text we have added to the manuscript in response is in ***green***. For Reviewer #3, our quoted previous responses are indented.

*

Reviewer #1: *The authors have done a good job of addressing most of my concerns from the first review. I do have a few relatively minor questions and comments.*

The biggest question still in my mind is that of biological plausibility. The biological plausibility (BP) of their method is a major theme of the paper, and is stated throughout, including the Abstract. For example, the second bullet point in the Highlights section states,

"Our hybrid predictive coding network jointly optimizes a single unified energy function by combining both iterative and amortized inference and is trained with biologically plausible prediction error minimization using only local Hebbian updates."

My main question, in this vein, is about how the amortized network is trained. According to [Bogacz, 2017], which is cited in the footnote in the 1st paragraph of section 2.3, biological plausibility adheres to two main criteria: (1) local computation, and (2) local plasticity. Local plasticity is described (in that paper) as the restriction that "Synaptic plasticity is only based on the activity of pre-synaptic and post-synaptic neurons." I agree that the predictive-coding part of HPC satisfies those BP criteria. However, it's not clear to me how the connection weights in the amortized network could be learned using a local plasticity (Hebbian) update rule. Since the authors are somewhat vague about how the corresponding error nodes are situated in their network (they excluded them from fig. 2), it is difficult to assess the validity of the BP claim.

Thank you for highlighting this ambiguity. Just as with the generative ("standard") PC network, the amortized network is learned using local Hebbain plasticity between prediction units and amortized error units, which compute the prediction error between layer L and L + 1 under transformation \phi_L (a "bottom

up" prediction). These errors can then be used to update the parameters \phi in a manner that adheres to the three BP constraints listed above. In this sense, the amortized network is just an "upside-down" generative PC network, and adheres to the same learning rules. We appreciate that this may not have been clear from Figure 2, and to help clarify this point, we have updated Figure 2 to make these error units explicit:

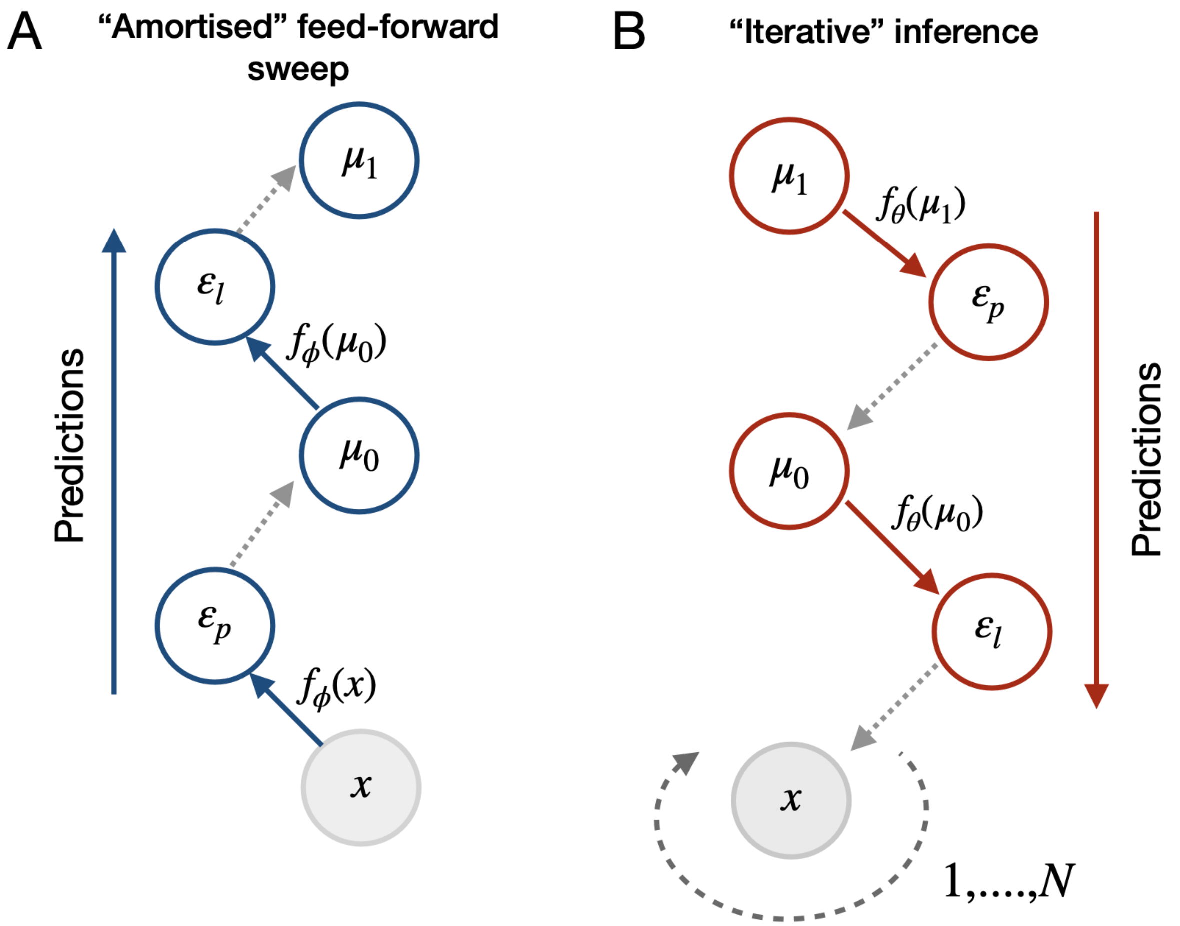

Moreover, we updated the text of Figure 2 to read:

These predictions are associated with error units that track the difference between variables at one level and the variables at the level above under transforms $f_{\phi}(\cdot)$. These errors are not utilised for inference but are used to update the amortised parameters ϕ during learning (weight updates).

The BP of weight normalization is another aspect of the HPC method that is unclear. In line 313, the authors state that they keep the mean of the amortization weights constant, but they don't say how. I raised this question in my first review, and the authors responded with "[weight normalization] may or may not be biologically plausible…", in addition to a reference to an arXiv paper. I think it would be prudent to specify which type of normalization they actually used. Not specifying, but making claims of BP throughout the paper suggests that they implemented weight normalization in a BP manner, which might be inaccurate, or even misleading.

We thank the reviewer for further expanding upon this point. To answer their query: the weight normalisation is computed by dividing the layer's weights by the mean of the layer, and is applied every time the weights are updated. Without the proposal of some additional mechanism, this weight normalisation is not strictly biologically plausible, as it requires information (the mean of the layer) that is not avaliable locally to each individual neuron. However, we do not believe that this directly negates claims of biological plausibility for the HPC network, as our experiments suggest that its inclusion has no percievable effect on the performance of the HPC network. Instead, the weight normalisation only has an effect on the standard PC results, as without the normalisation the weights grow large, thus causing performance to drop (this is discussed in the paper and work is referenced on line 411). This effect was not present in the other conditions, including our HPC network, however we included the normalization in all conditions to ensure fair comparison. We feel that to show poor performance on PC by excluding weight normalisation from all conditions would be highlighting an effect which distracted from the core points of the paper.

We have updated line 313 to read:

Weight normalisation, where the absolute values of the weights in a layer are divided by the mean of the weights of the layer, is used for the generative parameters θ, which we found crucial for maintaining classification accuracy in the standard predictive coding network.

On line 434, the authors mention "normalizing the values for the final layer to obtain an output probability distribution". Again, I would encourage the authors to be more explicit regarding the degree to which their implementation is BP. Moreover, what kind of normalization was used? Was it softmax, or some other type of scaling?

We thank the reviewer for highlighting this point. The normalization performed was simply dividing all values by the sum of the values. However, we note that this computation was performed to "read out" the models inferences for the purpose of plotting and measuring accuracy, and is not used in learning or or inference (in contrast to the weight normalisation referenced in the previous comment, which is used during model learning). In other words, we perform a series of post-hoc analysis (argument maxing to get the most likely hidden state, normalising to interpret the distribution of hidden states in terms of an entropy measure). These are computations performed "on" the network rather than intrinsic computations performed "by" the system. As such, we do not believe these computations need to be held to the same standard of biological plausibility. We hope this alleviates the reviewer's concerns on this particular point.

It is fine if parts of the algorithm are less biologically realistic than other parts. My main concern is about being transparent and precise about claims of BP. I would suggest the authors either describe in fuller detail how the amortized learning rule and normalizations are BP, or simply back away from those claims.

We agree and we hope that our changes clarify these issues about BP.

On line 358, they state "The accuracy of amortised inference increases at a slower rate, consistent with the intuition that learning discriminative models requires more data relative to generative models." However, the slower training of the amortized model could also be explained by the fact that it cannot learn until the PC network learns, since the amortized model is really just learning to predict the equilibrium state of the PC model. To me, this seems like a much more likely (and prosaic) explanation.

We thank the reviewer for highlighting this plausible explanation, this was an oversight on our part and we agree that the reviewers explanation of the effect is more likely - we have updated the text as follows:

This result is likely due to the fact that amortised inference is trying to predict the equilibrium point of iterative inference, which is changing as the parameters of the network converge.

On line 386, the authors refer to results that have not yet been presented; figure 5 seems to have the relevant data, but that figure is not referred to until the next paragraph. It seems that the paragraphs might have been swapped. Also, "iterative

iterations" seems redundant. Perhaps they mean "inference iterations".

Thank you for highlighting this error, we have moved the offending paragraph to 405, after the results are introduced, and updated "iterative iterations" to inference iterations.

We identify several unintuitive results that require further explanation, and which may be suitable predictions for empirical explorations. First, the accuracy of the amortised network improves slower as the number of inference iterations increases. We hypothesise that this is due to the targets for the amortised network being more difficult to predict (due to an increased number of iterations, leading to increased accuracy). Second, the amortised component can achieve high accuracy while the iterative component achieves low accuracy. This is due to the final layer loss - which is a function of the ground truth labels and which provides a learning signal in lieu of accurate iterative inference. Finally, we observe instabilities in the predictive coding network that occur at a low number of iterations, leading to a decline in accuracy. We hypothesize that these result from instabilities in the training procedure, which crucially are not found in the full hybrid network \citep{kinghorn2022preventing}.

With regard to MNIST, in my first review I questioned how many samples were in the training set, and said that I thought there were only 50,000. Looking into it further, it seems that the authors might have been correct in their first draft. I'm not too concerned with this particular detail, but just wanted to come clean on the issue. I trust the authors will know, from their own experiments, how many samples they trained on, and report that in the paper.

The reviewer was correct in their estimation of the test / train split and we have ensured that the text correctly states the size of the training set (50,000).

A natural, but unaddressed question is whether the HPC network converges to the same state every time. Does the initialization by the amortized network cause the iterative inference of the PC network to settle into a different final state than it would have if it was initialized differently? If so, under what circumstances?

This is a fascinating question and one which we have begun to explore in follow up work (namely, does a hybrid approach lead to *different* representations, rather

than just changing aspects of learning). We have included this open question in the discussion, on line 660:

An open question remains as to whether hybrid predictive coding learns \emph{different} representations, relative to standard predictive coding, or whether the observed effects just influence the manner in which the two networks learn. We hope to explore this question in future work.

Other comments:
- Line 391: The statement starting "Finally, the instabilities…" is not a sentence.

We thank the reviewer for catching this error and have updated the text to read:

Finally, we observe instabilities in the predictive coding network that occur at a low number of iterations, leading to a decline in accuracy. We hypothesize that these result from instabilities in the training procedure, which crucially are not found in the full hybrid network \citep{kinghorn2022preventing}.

- Equation (10): Should there be a prior probability factor included in the product, p(z_L)?

Thank you for highlighting this error, we have updated the equation to read, which includes the prior factor p(z_L):

p(\z, \x) = p(\x|\z_1)p(\z_1|\z_2)...p(\z_{L-1}|\z_{L})p(\z_{L})

- Line 164: The authors are comparing HPC to SPE. In the sentence, "In terms of technical content, the model's bidirectional connectivity is between individual neurons, rather than between layers". The authors should clarify that they are referring to the SPE model (I believe). Also, I don't think it's fair to say that the SPE model's connectivity is between neurons, and not between layers. Or, at least, it's not clear what is meant by that.

We agree with the reviewer that the terminology may have been confusing. The implication was HPC weights are updated based on layer-wise (a vector of activity) errors, whereas SPE was neuron-wise. We have removed the text as it did not pertain to the main differences between the model:

In terms of technical content, the update equations have the neuron's states continually updated based on bottom-up and top-down information, rather than the temporal asymmetry found in HPC.

- References: There are some typographical errors in the References section. In particular, proper nouns should be capitalized, like "Helmholtz", "Bayesian", "Hebbian", and "Kalman", as well as acronyms, like "VAE", and "FMRI". Also, I believe the arXiv paper [Millidge, Tschantz, Seth, Buckley, 2021] has been published, and that published version should be cited instead. Perhaps there are others.

We thank the reviewer for catching these errors, we have done a full review of the references and updated them accordingly.

In summary, this is an excellent paper that should be published once these rather minor wrinkles are ironed out.

*

Reviewer #2: *The authors' revision of their paper in response to my comments is sparse. Personally, I think the authors have missed an opportunity to increase the appeal of their paper to a wider audience by engaging with the comments in a bit more detail. But overall, they have addressed most of my comments satisfactorily.*

The only remaining issue I have is the discussion of the link between the model and phenomenology. In my view, this point remains relatively weak. I would urge them to consider being more specific and detailed in their discussion of this issue:

Comment 2 (in the authors' responses)

I think the authors either need to unpack the issue of how their model relates to phenomenology in far more detail – even if this point is speculative – , or remove it. If they wish to retain it, the authors need to explain in detail how the 'different correlates provide plausible accounts for their corresponding phenomenological properties.' In other words, which correlates correspond to which phenomenological properties, and how do they provide plausible accounts?

Though we have given it careful consideration, we are not quite sure how to respond to this point. We already state clearly in the introduction how top-down and bottom-up signals have been associated with distinct aspects of perception, highlighting accounts suggesting that both bottom-up and top-down signals contribute to perceptual content, but in different ways. We then note (p.5) that rapid gist perception is difficult to reconcile with the iterative processes underlying standard PC - implying that the contrast between detailed perception and gist perception is one aspect of phenomenology that our model could potentially shed light on. We return to this issue and make the above implication explicit in the Discussion (p.23 para 2), where we say "hybrid predictive coding provides a powerful computational lens through which to understand different forms of visual perception - in particular, the differences between fast context-free perception, such as gist perception, and slow, context-sensitive perception, such as detailed object recognition". In our view, this gives clear examples of aspects of phenomenology we are addressing, and the remainder of this paragraph explicates how HPC is relevant - as does the material in Section 4.2.

We of course recognise that our thoughts in these directions are preliminary and speculative, and do not pretend otherwise. We are keen to keep them, because we do believe they are relevant and will be of interest to the readership. However, to reinforce their speculative nature (it is beyond the scope of the paper to expand substantially on these points), we have added a line to the end of the first para in the Discussion (p.23)

"Further work under the banner of 'computational neurophenomenology' (e.g., Ramstead et al, Suzuki et al) is needed to examine this proposition in more detail, and in particular to explore how aspects of iterative/amortized inference correspond respectively to aspects of detailed/gist perceptual phenomenology."

We also soften the author summary to say

".. may also help shed light on the neurocomputational basis of some otherwise difficult-to-understand aspects of visual phenomenology" [ie replacing 'many' with 'some']

.. and we adjust the Highlights section, changing "potentially accounting for" to the softer "potentially shedding light on"

We emphasize that we do appreciate the reviewers point here, in that there is much more scope to develop these ideas. This is something we are determined to

do in future work

Comment 3 (in the authors' responses):

I don't think that the authors have responded to my comment, which might be my fault for not being clear enough. My point is that there is a tension between those theories of consciousness that emphasise recurrent processing as a key mechanism and the authors' HPC model. In my view, if the authors wish to retain the comments linking HPC to phenomenology, then they need to detail how their account relates to theories of consciousness that emphasise recurrent processing. This is important because, as far as I understand, the authors claim that the specific characteristic of conscious phenomenology is determined by the balance between the amortized and the iterative components, while theories of recurrent processing emphasis the recurrent component exclusively.

It is true that some theories of consciousness emphasize recurrent (i.e., top-down) signalling as key for conscious perception (Lamme et al. 2000, Pascual-Leone et al 2001, Seth & Bayne, 2022). However, the experimental evidence favouring such theories does not (to our knowledge) discriminate the forms of perception discussed in our study: e.g., detailed vs gist perception. We agree it is worth saying something more though, so we have added (as a new paragraph at the end of Section 4.2):

"Interestingly, some prominent theories of consciousness emphasize recurrent (i.e., top-down) signalling as critical for conscious perception (Lamme et al. 2000, Pascual-Leone et al 2001, for a review see Seth & Bayne, 2022). While these theories are not always interpreted in terms of perceptual inference, it is natural to do so (Seth, 2021). This however raises an interesting possibility: could it be that conscious perception depends not on top-down signalling defined anatomically (as in standard recurrent theories) but, instead, defined functionally, in terms of their role in conveying predictions (as opposed to prediction errors)? This would predict that the relationship between conscious status and (anatomical) top-down signalling would depend on the details of the perceptual task and/or aspect of phenomenology being assayed. Future work could usefully investigate this possibility."

New refs:

Ramstead et al: https://link.springer.com/article/10.1007/s13164-021-00604-y

Suzuki et al: https://www.nature.com/articles/s41598-017-16316-2

Lamme, V. A., Super, H., Landman, R., Roelfsema, P. R. & Spekreijse, H. The role of primary visual cortex (V1) in visual awareness. *Vis. Res.* **40**, 1507–1521 (2000)

Pascual-Leone, A. & Walsh, V. Fast backprojections from the motion to the primary visual area necessary for visual awareness. *Science* **292**, 510–512 (2001).

Seth & Bayne: https://www.nature.com/articles/s41583-022-00587-4

Reviewer #3*: My review is uploaded as an attachment. (Text transposed here)*

In this revised manuscript, Tschantz et al. partly address the concerns I raised in my previous review. I thank them for the revisions they have made, and I want to stress that I remain very enthusiastic about the work in general, and especially the ideas behind it. However, I also still have strong reservations about some aspects of how the work is presented. These reservations concern (1) a misrepresentation of the role of bottom-up connections in "vanilla" predictive coding models as compared to the HPC model, (2)misguided and misleading (and, indeed, unnecessary) claims about the (putative) probabilistic or variational nature of the HPC model's computations and representations and (3) a misrepresentation of results regarding data efficiency. While these concerns

can be addressed with rather minor revisions or additional analyses, I do find them to be of high importance for the paper's scientific soundness.

For clarity, I have explained my concerns in detail below, as responses to the replies made by the authors to my earlier points.

Comment 1

> ***We thank the reviewer for pointing out this clarification - we have clarified that the role of bottom-up (and top-down) connectivity and activity distinguishes the two frameworks. We have added the following text to the caption of Figure 1:***
>
> ***Although there is bottom-up information flow [in PC] in terms of errors, this bottom-up information only conveys errors. However, in our hybrid predictive coding models, bottom-up information conveys both predictions and errors (along with top-down information).***
>
> ***We have added the following text to line 117:***
>
> ***This contrasts with traditional predictive coding, where bottom-up signals only convey errors, and bottom-up connectivity does not implement transformations between layers (i.e. does not implement predictions).***

I appreciate these revisions, and I feel that figure 1 is now sufficiently clear. However, I still have reservations about figure 2 and the description in the text.

In their revised text, the authors write that, in traditional PC, "bottom-up connectivity does not implement transformations between layers" and "bottom-up signals only convey errors". But said errors of course are multiplied by synaptic weights in order to drive updates to the representation units in the layer above. This multiplication (and subsequent summation) surely constitutes a transformation, and this is fundamentally no different from the bottom- up transformations that occur in the amortized component of the hybrid PC model. In the latter, the activities of the representation units are multiplied by synaptic weights to directly yield a prediction of the activities of the representation units above. Both constitute a linear transformation of an activity vector (combined with a pointwise non-linearity). So, the difference is not that one is a transformation while the other isn't – the difference is in what each transformation accomplishes. One projects prediction errors onto the Jacobian of the (local) generative model, while the other projects the mu's onto an approximate inverse of that generative model. In both cases, the bottom-up synaptic weight matrix has the role of transforming a lower-level representation into a higher, more abstract one. Thus, the current phrasing

is doing a disservice to traditional PC models, and does not accurately explain the contribution of the amortized bottom-up stream that is added in the HPC model.

We thank the reviewer for this thoughtful and accurate point. We have updated the text of Figure 1 to read:

Although there is bottom-up information flow [in PC] in terms of errors, this bottom-up information conveys errors (transformed by the synaptic weights). However, in our hybrid predictive coding models, bottom-up information conveys both predictions and errors (along with top-down information).

And we have updated line 117 to read:

This means that [in HPC] both bottom-up and top-down signals convey transformations of errors and transformations of activity. This contrasts with traditional predictive coding, where bottom-up signals convey a transformation of errors while top-down signs convey a transformation of activity (predictions).

As for figure 2, my issue with this is that there are no bottom-up arrows from the prediction errors to the mu's in the layer above, and I really feel there should be (especially since there are arrows from the errors going down to the mu's in the layer below, even though these arrows represent the same functionality and connectivity that is omitted in the bottom-up direction). In its current design, the figure incorrectly suggests that the iterative inference is purely top-down, and that errors only drive updates to the layer directly below them.

We thank the reviewer for taking time to expand on this point, and are in agreement that our current treatment glosses over some of the complexity involved in bottom-up signalling in standard PC. We have updated Figure 2 (attached below) to indicate the existence of "bottom-up" / "amortised" errors which track the error between layer L and L + 1 under the transformation phi_L.

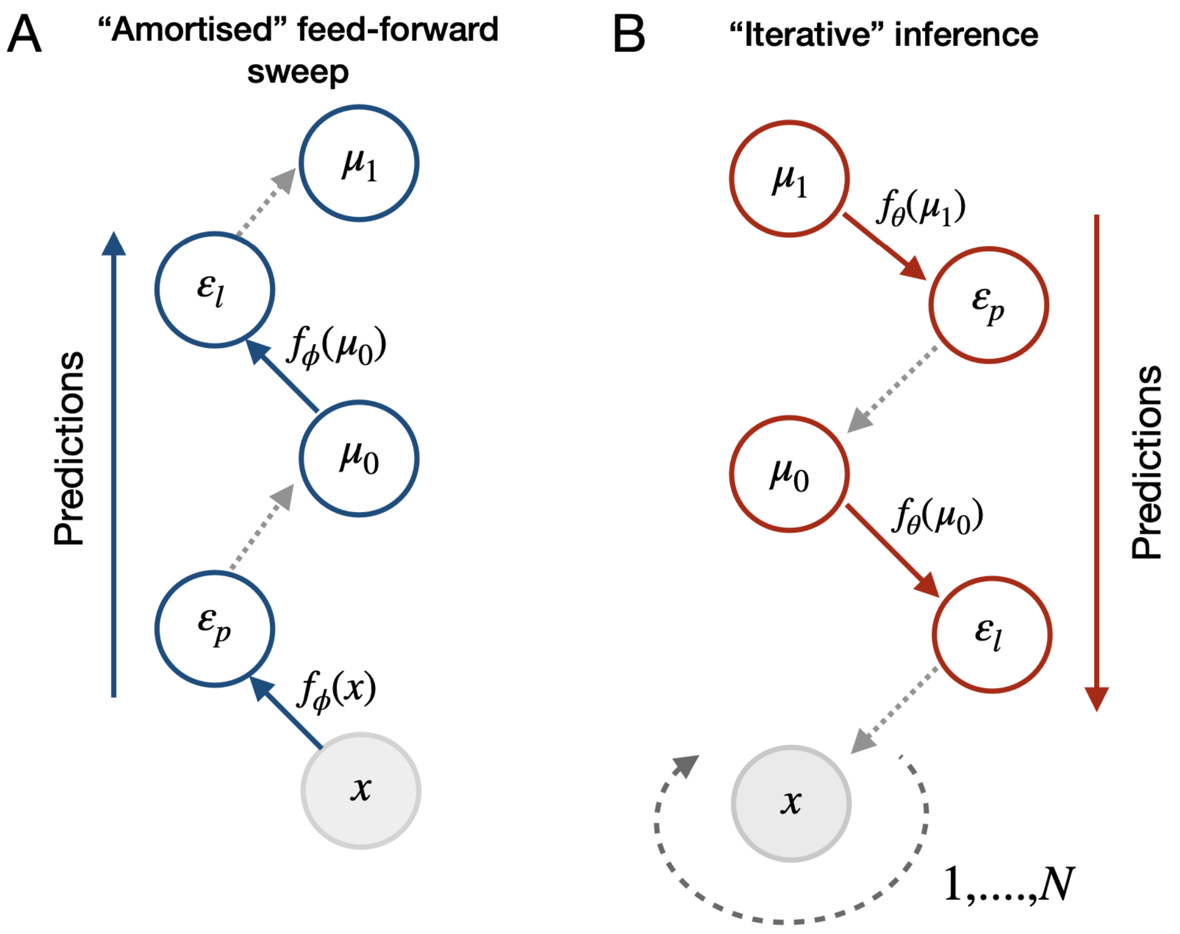

And we have updated the text of Figure 2 to read:

These predictions are associated with error units that track the difference between variables at one level and the variables at the level above under transforms $f_{\phi}(\cdot)$. These errors are not utilised for inference but are used to update the amortised parameters ϕ during learning (weight updates).

Comment 4

We thank the reviewer for their insightful comment. The purpose of this experiment is to compare the relative times to asymptote, rather than absolute performance. Thus a stopping criterion is used for when one of the two networks stops improving. We explore asymptotic performance in Figure 3a, where we explore the asymptotic performance of the networks (i.e., run them all to convergence). Indeed, as predicted, the amortised

predictions do reach the full accuracy of the predictive coding and hybrid predictive coding networks.

If the purpose was to compare the speed of training, then this analysis does indeed make more sense. However, speed of training (or time to asymptote) is not a measure of data efficiency – a model can train very slowly and yet be very data-efficient. More to the point, if the amortized component of the HPC model eventually converged to a similar performance as the full HPC model, then both would be equally data-efficient. So, at present, the claims made on this point (e.g., "These results show that incorporating a top-down, generative component substantially ***increases data efficiency****." (l. 426-427)) are not substantiated by the data. The simplest way to address this concern would be (as I suggested previously) to compare asymptotic performance in the low-data regime. If the authors are unable to do this comparison, then I feel they should revise the claims made in the manuscript to be about speed of training, rather than data efficiency,*

In addition, if the authors prefer to stick with an analysis of training speed, then I think they need to revise some of their phrasing in the text, which implies that their present results constitute a comparison of accuracy per se. For instance (emphasis mine):

Thank you for clarifying this point, this was an omission on our end. We have elected to revise claims in the paper to reflect the fact fact that our results demonstrate an increased speed of learning, rather than data efficiency per se. We have included the updated text below, with the original text indented:

> *"Here, we demonstrate this data-efficiency by plotting accuracy as a function of dataset size. Specifically, we compare the accuracy of hybrid predictive coding compared to the amortised predictions using datasets with 100, 500, 1000 and 5000 examples" (l. 420-422)*

Here, we demonstrate the speed at which the network learns by plotting accuracy as a function of the amount of data the network has been exposed to.

> *"Figure 6: Accuracy as a function of dataset size. (A) 100 examples. The accuracy of hybrid predictive coding is lower than with the full dataset, but still high given the minimal amount of data (0.17 percent). The accuracy of the amortised predictions is significantly worse." (Fig. 6 caption)*

The accuracy of hybrid predictive coding is lower than with the full dataset, but still high given the relatively small amount of data the network has been exposed to …

Importantly, the poor performance of amortised inference at the start of learning does not negatively impact the speed at which iterative inference learns…

We have updated line 422 to read:

These results show that incorporating a top-down, generative component substantially increases the speed at which the network learn

Comment 5

> ***We take the point that predictive coding, in the current setting, shares many features with a deterministic system. However, we prefer to keep the current terminology as it is ubiquitous in the predictive coding literature to describe it as probabilistic due to the fact it is motivated by and derived from variational inference. The point mass distributions on the approximate posterior can be motivated by the Laplace approximation [1]. Moreover, the likelihoods are still treated as Multivariate Gaussian distributions, albeit with fixed second-order statistics. We have added the following text to the manuscript to clarify (line 212)***
>
> ***It is for this reason that predictive coding is often considered a `probabilistic` theory of learning (as the model variables can be read as sufficient statistics of posterior beliefs), even when all of the distributions are point mass or with fixed second-order statistics.***

I do not feel that this addition addresses my concern, and I disagree with the authors' reasoning and assertions.

Predictive coding was not originally derived from variational inference, and does not uniquely derive from this framework. As the authors are no doubt aware, one can derive predictive coding simply from a mean-squared-error (MSE) loss, or from maximum-likelihood (ML) or maximum-a-posteriori estimation (MAP) on a hierarchical Gaussian generative model. Optimizing a MSE loss can be seen as a special case of ML or MAP estimation (by assuming IID Gaussian noise at all levels, combined with a flat prior), just as ML and MAP estimation can in turn be seen as a special case of variational inference (if the variational beliefs are set to be point masses). However, while these connections are insightful, it is misleading to refer to each special case by its parent category, just as it would be misleading to refer to a parabola as a 10-D polynomial (even though the former is technically a special case of the latter). Using the more general label implies the additional properties that distinguish the special from the general case (for lack of them).

PC models can, and have been, extended or modified to perform variational inference – that is, to represent variables by non-trivial probability distributions that approximate the true posterior. But HPC is not such a model, and PC models are not variational by default. (There is a reason why VAEs are called "variational" in contrast to regular autoencoders. By the logic put forth by the authors, all autoencoders would be VAEs, and indeed, all linear regression analyses would be variational too, and so on.)

The Laplace approximation can of course be used to estimate an approximate posterior around a MAP estimate, but the method presented here does not employ such an approximation. So, just as one should not market an automobile as an aircraft because one could affix wings to it, the HPC method should not be called "probabilistic" merely because one might combine it with a Laplace approximation.

To clarify, I have no objections to calling the generative model "probabilistic", to pointing out that PC can be thought of as performing inference on a PGM, or to explaining the relationship between (H)PC and variational inference. However, to refer to the representations computed by the HPC inference process (or that inference process itself) as "probabilistic", or to refer to any aspect of the HPC model as "variational" (e.g., "variational iterations"), is to stretch these terms beyond their breaking point, and to imply that the model has abilities that it does not possess.

(See also Ma, W. J. (2012). Organizing probabilistic models of perception. Trends in cognitive sciences, 16(10), 511-518.)

Thank you for taking the time to state this point in detail. We have updated the language throughout the text so that notions of probability distributions and variational inference are only mentioned (a) when discussing the generative model or (b) providing background motivations, e.g. in terms of highlighting the relationship between HPC and variational inference. We have removed several instances where the process and representations involved in HPC are referred to as probabilistic or variational.

Line 391 (removed variational):

Hybrid predictive coding can obtain equivalent performance with a little as 10 iterations (Figure \ref{fig:5}A)

Line 387 (removed 2 mentions of variational):

Another interesting phenomenon is the relative difference in accuracy between the full hybrid model and its amortised component as a function of iterations.

When the number of iterations is lower (e.g., Figure \ref{fig:5}A), the relative difference between these accuracies is far less pronounced since the accuracy of the pure iterative inference predictive coding network is unstable and decreases over time when there are an insufficient number of inference iterations.

Line 492 - probabilistic beliefs to representation

Moreover, by providing a rapid initial `best guess' at \edit{representations} \citep{bar2003cortical, kveraga2007top, kreiman2020beyond}, amortised inference provides informed initial conditions for the subsequent phase of iterative inference,

Line 501 - removed reference to probabilistic beliefs

Our model also naturally balances top-down and bottom-up contributions to perceptual inference. The degree to which bottom-up and top-down predictions contribute to the final beliefs

Line 505 - removed reference to probablistic

By combining amortised and iterative inference within a single model, the resulting scheme naturally facilitates an adaptive trade-off between speed and accuracy,

Line 283 - removed reference to probablistic

While there have been several proposals for how feedforward and recurrent activity may be integrated in the brain, our model is the first to combine these into a common and biologically plausible predictive-coding architecture.

Line 583 - removed reference to probablistic

Errors will be large and iterations of recurrent activity are engaged to finesse beliefs. Crucially, this arbitration arises naturally from the representations within the model. In summary, our model provides a plausible account for both feedforward and recurrent activity in the brain, which can be related to distinct forms of visual perception.

Line 106 - removed reference to probablistic

Thus, amortised inference provides a plausible mechanism for extracting beliefs rapidly and efficiently via feed-forward, bottom-up processing \citep{gershman2014amortized, van2020going}.

Line 109 - removed reference to probablistic predictions

Our novel neural architecture - \emph{hybrid predictive coding} (HPC) - combines (standard) iterative predictive coding with amortised inference, so that \emph{both} bottom-up \emph{and} top-down signals convey predictions (and where prediction errors also flow in both directions) \edit{(see Figure \ref{fig:1})}.

Figure 1 - removed reference to probabilistic

Our ``fast and slow'' model casts this integration of bottom-up and top-down signals in a common framework, allowing derivation of a testable process theory

Comment 7

> ***We thank the reviewer for raising this extremely relevant point. We believe that the requested data can be determined from the existing manuscript and figures. Directly after the distribution shift, we run inference (and thus no learning has happened since the distribution shift). The point on the x-axis directly after the distribution shift, therefore, signifies the number of iterative iterations, which jump from ~1 before the shift to ~100 after the shift, with no further retraining (for that particular point). The rest of the graph shows how the number of iterations decreases as the system fits the new distribution. We believe that this is different from the amortization gap, which would simply show the final loss before and after the distribution shift, rather than the number of iterations employed. Indeed, after the distribution shifts the network does fail to classify (not shown in figures), resulting in larger prediction errors after the amortized sweep, which incurs more recurrent processing. This figure is helpful to consider in combination with 7b, which shows that vanilla predictive coding does not induce significantly reduced iterations as a function of learning (due to the network being randomly initialized for each stimulus) - so the number of iterations does not track familiarity with the data distribution.***

Thank you for providing this explanation. I think I am beginning to understand, although it would be helpful (also to other readers, I suspect) if you could fill in a few more gaps for me. My confusion stemmed from the assumption that the network should never reach the threshold level of perceptual certainty, when faced with a digit from an unfamiliar class (e.g. an ‘8’ when only 0-4 were seen during training). However, I now surmise that this intuition was incorrect, and that the network nevertheless converges to some sufficiently confident (but most likely incorrect) guess about the digit class. Is this understanding correct? I guess this must depend on the choice of threshold: with a

sufficiently low threshold, I would expect my initial prediction to be confirmed (at least in some instances). Perhaps it would be useful to plot the classification accuracy along the number of iterations, to show that there is nothing "magical" going on (i.e. the model cannot classify digit classes it never saw before).

I think this is worth noting because it means that, while the network is able to converge to some answer with sufficient iterations, no amount of iterative processing is sufficient to accurately classify digits from unfamiliar classes.

My confusion was also specifically fed by the following passage:

"As 7C shows, this dramatically increases the number of iterations required to reach perceptual certainty. This is because the bottom-up, amortised predictions have not learned to predict the relevant model variables, leading to a poor initialisation and an increase in prediction error."

I would have thought that the top-down predictions would be just as inaccurate (at least at higher levels of representation, nearer the digit class units) as the bottom-up, amortized predictions. After all, the network has not (if I understand correctly) had the chance to learn a generative model of the unseen digit classes (though these digits of course share lower-level features in common that will have been learned). Is this not the case, or does this not contribute to the increased number of required iterations?

(The wording of this passage initially led me to think that perhaps the generative model (i.e. the top- down connections) had been trained on the withheld digit classes, thus enabling the iterative inference to accurately classify the digit, only without the benefit of a good initialization.)

We thank the reviewer for expanding on this point, and agree that the relationship between the number or iterations and classification accuracy, as well as the contributions of bottom-up and top-down signals, requires further clarification. We respond to the reviewers questions and then provided the updated text below.

The reviewer is correct in their intuition that "*no amount of iterative processing is sufficient to classify unfamiliar classes*". Indeed, the relationship between prediction errors and the final accuracy of beliefs is non-trival; you could achieve zero prediction error for a randomly initalised network (which might be expected if the number of hidden units is sufficiently large), or high prediction error for a converged network that achieves accurate beliefs. Therefore, perceptual certainty should not be thought of as an objective measure of belief accuracy, but rather as a metric that tracks the speed at which the network converges to equilibrium. We

have added a paragraph that highlights this distinction and mentions the results requested by the reviewer.

In reference to the second point, the reviewer is correct that the top-down, generative network also contributes to the increased number of iterations when the data distribution shifts. This is because, as the parameters of the standard PC network are learned under a fixed data distribution, the number of iterations required to go from random initialization to the equilibrium point of the network generally decreases (as the learning changes the landscape that the iterative process operates on). This effect is demonstrated in Figure 7B (orange line), where the number of iterations required to achieve "perceptual certainty" decreases in standard PC, albeit at a much slower rate.

We have updated the text so that it is explicit that the increased number of iterations in Figure 7C is (a) a measure of how long the network takes to converge, rather than a measure of classification accuracy and (b) this is a result of both the amortized network no longer being able to predict the equilibrium point of the network (due to the fact it has changed), and the landscape induced by the parameters of the generative network.

Line 459 now reads:
Crucially, the relationship between prediction errors and the final accuracy of the beliefs is non-trivial; it is possible for a randomly initialised PC network to achieve zero prediction error in a short number of iterations, and a fully converged PC network to incur a large amount of prediction error after several iterations, while still achieving accurate beliefs. This is demonstrated in experiment Figure \ref{fig:7}C, where directly after the change in data distribution, the HPC model cannot classify the previously unseen digits but still manages to achieve perceptual certainty after several iterations. In this context, perceptual certainty reflects to the equilibrium point of inference, which may or may not reflect accurate beliefs. As such, this plot tracks the number of iterations required to go from the initial state to the equilibrium state of the network. As demonstrated in Figure \ref{fig:7}B, this is mostly facilitated by the role of the amortised network, which provides an initial state closer to the final equilibrium. However, it is partly facilitated by learning the parameters of the generative network (orange line), as learning changes the optimisation landscape that the iterative process operates on, enabling activity to converge in fewer iterations.
